# Vaccine safety in Australia during the COVID-19 pandemic: Lessons learned on the frontline

**DOI:** 10.3389/fpubh.2022.1053637

**Published:** 2022-11-04

**Authors:** Ingrid Laemmle-Ruff, Georgina Lewis, Hazel J. Clothier, Gerardo Luis Dimaguila, Michelle Wolthuizen, Jim Buttery, Nigel W. Crawford

**Affiliations:** ^1^SAEFVIC, Infection and Immunity, Murdoch Children's Research Institute, Melbourne, VIC, Australia; ^2^Melbourne School of Population & Global Health, Faculty of Medicine, Dentistry and Health Sciences, The University of Melbourne, Melbourne, VIC, Australia; ^3^Centre for Health Analytics, Melbourne, VIC, Australia; ^4^Vaccine Safety & Evaluation, COVID-19 Response, Department of Health, Victorian Government, Melbourne, VIC, Australia; ^5^Department of Paediatrics, The University of Melbourne, Melbourne, VIC, Australia; ^6^Immunisation Service and General Medicine, Royal Children's Hospital, Parkville, VIC, Australia

**Keywords:** vaccine safety, pharmacovigilance, surveillance, immunisation, COVID-19 vaccines

## Abstract

Surveillance of Adverse Events Following Vaccination in the Community (SAEFVIC), Victoria's vaccine safety service for reporting adverse events following immunisation (AEFI), has provided integrated spontaneous surveillance and clinical services for individuals affected by AEFI since 2007. We describe SAEFVIC's response to the COVID-19 vaccine program, and reflect on lessons learned for vaccine safety. The massive scale of the Australian COVID-19 vaccine program required rapid adaptations across all aspects of SAEFVIC's vaccine safety services. Collection of AEFI reports was streamlined and expanded, incorporating both spontaneous and active surveillance data. Dramatically increased report volumes were managed with additional staffing, and innovations to automate, filter, and triage reports for priority follow up. There were two major adverse events of special interest (AESI): thrombosis with thrombocytopaenia syndrome and myocarditis, with multiple other AESI also investigated. Rapid escalation mechanisms to respond to AESI were established, along with AESI-specific databases for enhanced monitoring. Vaccine education and training resources were developed and public-facing vaccine safety reports updated weekly. Frequent communication with local and national government and regulatory bodies, and consultation with specialist groups was essential. The COVID-19 vaccine program has highlighted the importance of vaccine safety in supporting public confidence in vaccines and informing evidence-based immunisation policy. Supporting the COVID-19 vaccine program has required flexibility in adapting to policy changes and evolving vaccine safety signals, careful triage and prioritisation, informatics innovation, and enhanced engagement with the public regarding vaccine safety. Long-term investment to continue strengthening vaccine safety systems, building on lessons learned, will be essential for the ongoing success of Australian vaccination programs.

## Introduction

Post-licensure vaccine safety surveillance plays a key role in supporting public confidence in vaccines and informing evidence-based immunisation policy (1). Vaccine safety systems aim to facilitate early detection and investigation of adverse events following immunisation (AEFI), regardless of causality, to enable an appropriate public health response (2). The COVID-19 vaccine program has placed vaccine safety in sharp focus, creating significant challenges, and also opportunities to strengthen vaccine surveillance systems (3).

Vaccine pharmacovigilance systems vary globally, depending on available resourcing and healthcare systems, but most commonly include a spontaneous AEFI reporting component (2). In Australia, AEFI reporting occurs through widely differing jurisdictional-based spontaneous (passive) surveillance systems (4). All AEFI reports are forwarded to the national regulator, the Therapeutic Goods Administration (TGA), who has responsibility for monitoring the safety of all vaccines approved for use in Australia (5). Adverse events following immunisation reports can also be made directly to the TGA, who then refer reports back to jurisdictions for further review and clinical follow-up as indicated.

**Figure 1:**
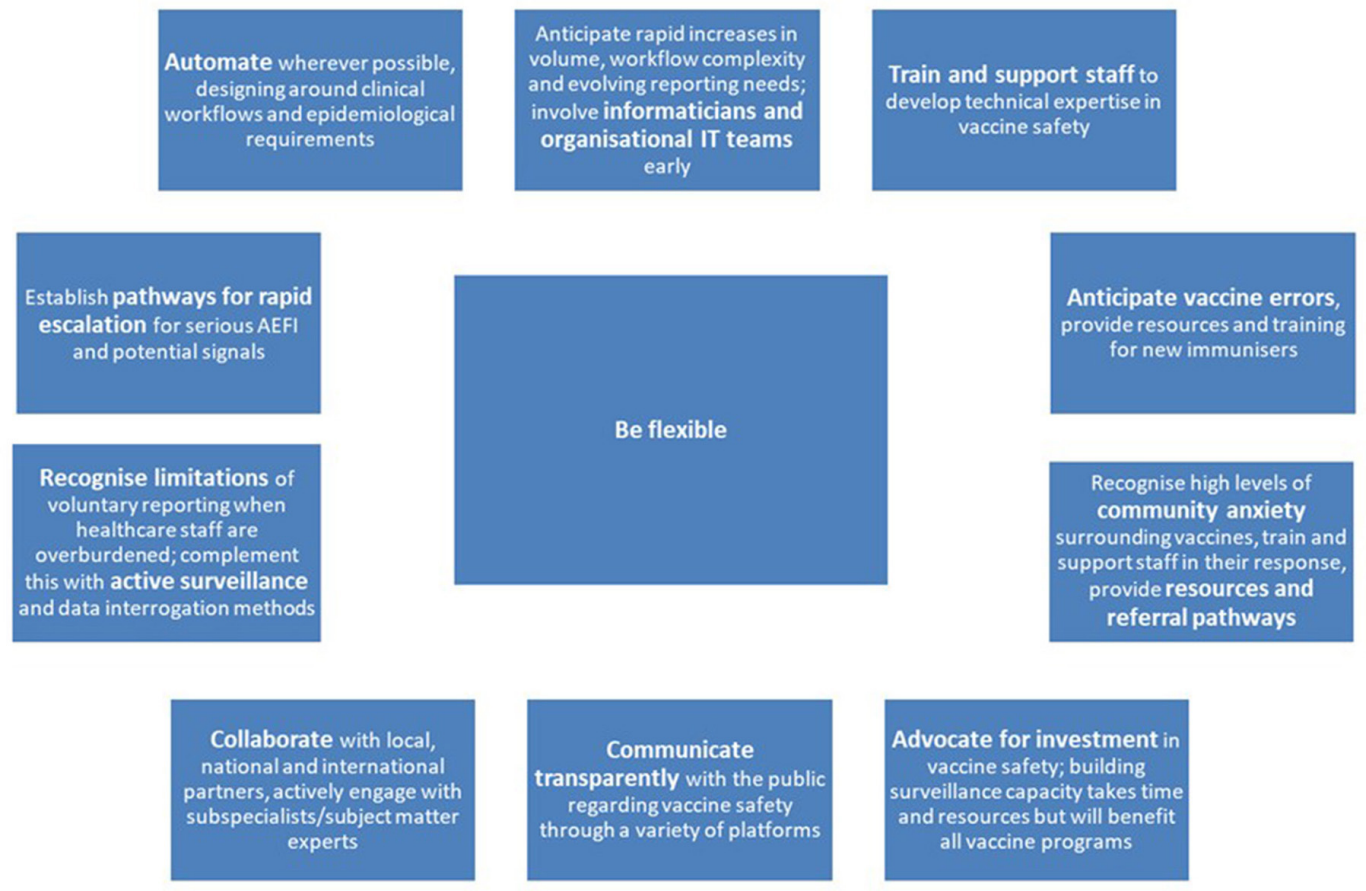
Lessons in vaccine safety during the COVID-19 vaccine program.

Surveillance of Adverse Events Following Vaccination in the Community (SAEFVIC) began in 2007, and is the vaccine safety service for the state of Victoria, Australia (population ~6.5 million, ~25% of Australian population) (4, 6). Funded by the Victorian Department of Health, SAEFVIC provides integrated enhanced spontaneous surveillance and clinical services, across all vaccine types administered in Victoria (7). SAEFVIC currently contributes 38% of all AEFI reports received by the TGA, and is the lead jurisdiction by AEFI reporting volume nationally (8). Enabled by the support of the Victorian Department of Health, SAEFVIC's adaptations over 2020–2022 have been significant, spanning all aspects of vaccine safety. This paper describes the key elements of SAEFVIC's response to the COVID-19 vaccine program up to 31 July 2022, and reflects on lessons learned for vaccine safety (Figure 1). These learnings and recommendations are relevant both for current vaccine safety activities, and in preparing for future vaccine programs, including those linked to future pandemics.

**Figure 2:**
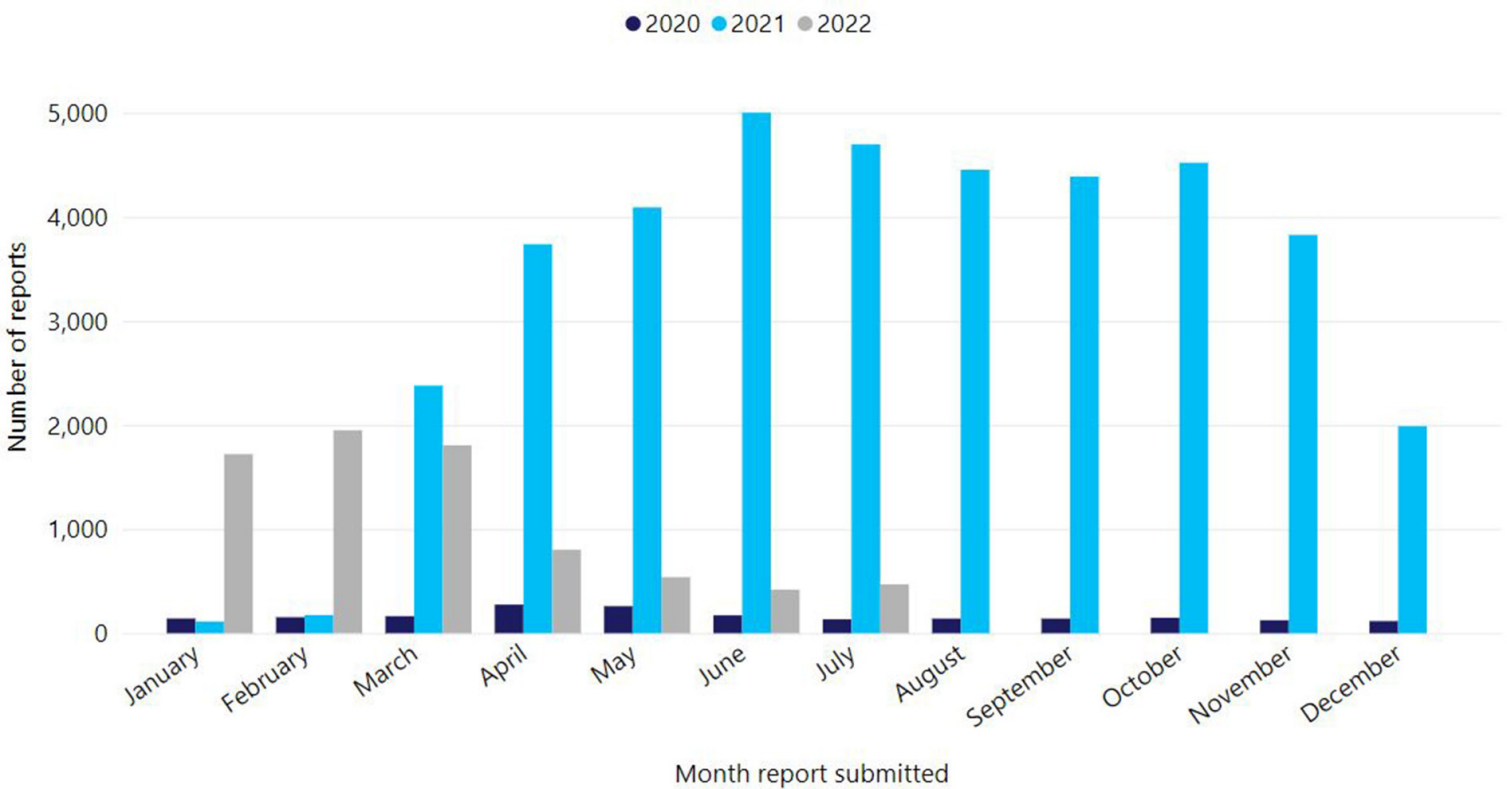
Reports of adverse events following immunisation for all vaccines, Victoria, 1 Jan 2020–31 July 2022.

Victoria's COVID-19 vaccination program rollout began on 22 February 2021. Up to 31 July 2022, 16,316,981 COVID-19 vaccine doses have been administered in Victoria (68%, 11,171,729 Comirnaty, 23%, 3,829,394 Vaxzevria, 8%, 1,276,013 Spikevax, and <1%, 39,845 Nuvaxovid) (8). Victoria's primary course coverage is high with 94.4% of people aged ≥16 years having received at least two primary doses, as well as high coverage rates for booster doses in those over 65 years (92.9% with three doses, 60.1% with four doses) (9). However, like many countries, primary two dose coverage in children 5–11 years has been modest (43.4%) (9, 10). Given the scope of the COVID-19 program, AEFI report volumes increased precipitously (Figure 2). In 2021 alone, there was a 20-fold increase in AEFI reports compared to previous years, with 96% (37,695/39,368) of reports following COVID-19 vaccines. Up to 31 July 2022, SAEFVIC has received 44,626 AEFI reports overall following COVID-19 vaccines (8). Notably, 909 episodes of COVID-19 vaccine-related error were reported, with a single error report often affecting multiple vaccines. Common errors reported included vaccine administration outside recommendation (e.g., incorrect dose, incorrect interval, incorrect age, incorrect vaccine), administration of expired vaccine, and less commonly, vaccine handling errors such as cold chain breaches.

Follow up of Adverse Events of Special Interest (AESI) has been a key component of the vaccine safety response. The AESI for COVID vaccines have been categorised internationally by the Brighton Collaboration (11). They include two major safety signals; thrombosis with thrombocytopenia syndrome (TTS) and myocarditis that required detailed investigation, which SAEFVIC contributed to at a state, national, and international level. Up to 31 July 2022, SAEFVIC has received reports of: 53 cases of TTS, 326 cases of myocarditis, 1,016 cases of pericarditis, as well as 29 cases of Guillain-Barré Syndrome following COVID-19 vaccines in Victoria (8). Additionally, five deaths in Victorians have been assessed as likely vaccine-attributable by the TGA commissioned Vaccine Safety Investigation Group using World Health Organisation causality criteria (12). These deaths each occurred from a different AESI: TTS, Guillain-Barré Syndrome, acute disseminated encephalomyelitis, a rare neurological condition related to transverse myelitis and myocarditis; four followed the Vaxzevria vaccine and one followed the Spikevax vaccine (13).

## Vaccine safety during COVID-19 vaccine program

### Collection of AEFI reports

As described, SAEFVIC operates a system of enhanced spontaneous surveillance (7). AEFI reports can be submitted online or by phone by healthcare providers, families, and/or patients. Families and patients had been confirmed by SAEFVIC to report a similar proportion of serious AEFI as immunisation providers (14). Prior to the COVID-19 vaccine program, immunisation nurses reviewed all reports, and specialist immunisation consultation was offered to individuals or families of children experiencing significant AEFI or with concerns. This system has continued throughout the COVID-19 program, with important adaptations described in the next section. Additionally, SAEFVIC Rapid Reporting was introduced in mid-2021, allowing streamlined reporting of common or expected side effects to contribute to data collection, without linking to clinical follow up.

Spontaneous surveillance is useful for signal detection, but underestimates rates of AEFIs due to under-reporting (2). Reliance on spontaneous reporting for serious AEFI became particularly problematic in the context of busy and over-stretched health-care staff in hospitals and primary care, with limited capacity to add AEFI reporting to their already heavy workload during the pandemic. Active surveillance, whilst often more resource intensive, can enable earlier and more sensitive detection of AEFI, and has provided an important mechanism to strengthen surveillance during the pandemic (2, 15, 16). SAEFVIC already contributed to the Paediatric Active Enhanced Disease Surveillance network, a hospital-based active surveillance system monitoring serious potential AEFI in children (17). In collaboration with the Victorian Department of Health, active surveillance activities were expanded substantially over 2021–2022, to include solicited surveillance and syndromic surveillance from multiple alternate data sources, as well as vaccine safety data-linkage.

For example, AusVaxSafety, a national active vaccine safety surveillance system, followed up vaccinees with brief SMS message surveys on days 3, 8, and 42 following COVID-19 immunisation (18). During 2021, AusVaxSafety expanded participating sentinel primary care sites in Victoria, and the SMS message surveys were also incorporated into the Victorian Government's COVID-19 Vaccination Management System servicing the state-based vaccination hubs (As COVID-19 vaccination has moved to the primary care setting, these surveys are now sent only from sentinel participating practices in Victoria.). A proportion of these survey responses, filtered for relevance (see next section), were integrated into SAEFVIC's AEFI reporting database. Consequently, the contribution of active surveillance was 23.6 times higher in 2021 during the peak of reporting (47.5% of all reports compared to an average across the preceding 5 years of 2.0%). Up to 31 July 2022, close to half (46.1%, 20,554/44,626) of AEFI reports following COVID-19 vaccines have come from active surveillance sources.

Further, this has been complemented by work analysing de-identified surveillance data from emergency departments, general practice clinics, Nurse-on-call telephone advice calls, residential aged care facilities, and social media, providing additional mechanisms to monitor AEFI trends and detect signals.

### Managing workflow and analysis of reports

The dramatically increased numbers of AEFI reports following the introduction of COVID-19 vaccines necessitated changes to SAEFVIC's workflow and management of reports (see Supplementary materials) (19). These changes required additional staffing, as well as informatics innovations to automate, filter, and triage reports for priority follow up. Given a large influx of phone and email queries, phone line options were refined, autoreplies with links and FAQs were developed, and an internal ticketing system was implemented, enabling a quick response and resolution of common queries. Of note, high levels of community stress and anxiety surrounding COVID-19 vaccines were reflected in phone calls and correspondence received by SAEFVIC. Training and supporting SAEFVIC's staff in responding to these kinds of enquiries, with response scripts, onward referral options, and structured pathways for escalation became important.

A priority triage list of AEFI reports received within the AEFI reporting database was developed and reviewed by nursing staff daily, using coding and key word analysis to identify reports requiring prompt follow up. Clinically-relevant natural language processing filters were placed on the COVID-19 Vaccination Management System survey response data, to restrict inclusion to medically-attended adverse events and minimise the need to review more minor expected AEFI reports. Procedures for report coding required regular updating, as new Brighton Collaboration criteria for specific AESI emerged, and vaccine eligibility criteria changed. Forwarding of AEFI reports to the TGA was streamlined, with commencement of a mapped, automated daily upload of new and amended reports.

SAEFVIC's processes for signal detection involve a combination of reactive response to clinical suspicion and queries (often flagged by SAEFVIC's nurses) and proactive analysis of AEFI reports for trends (4, 7). These processes were expanded during the COVID-19 program, with rapid cycle analyses regularly run to check for signals across AEFI reports. Data processing workflows were scaled up to manage additional external data sources such as jurisdictional immunisation records, for calculation of AEFI rates. Both cloud and in-house technologies were utilised to enable secure, efficient development of epidemiological AEFI reporting methods.

Despite the many modifications made, keeping up with backlog and adapting the existing database to rapid and substantial increases in AEFI reports remains a challenge, and ongoing work is occurring to continue to improve SAEFVIC's data management systems.

### Response to AESI and serious AEFI

For AESI and serious AEFI, pathways for liaising with treating clinicians and obtaining medical records were expanded, with support from the Victorian Department of Health's Vaccine Safety and Evaluation team. Rapid escalation mechanisms were established to discuss significant cases and signals of interest, and plan appropriate action. An on-call clinician roster allowed for after-hours medical advice where necessary. Alert Advisory Group (AAG) meetings became an important forum, chaired by the Department of Health and including treating clinicians, consulting specialists, epidemiologists, and vaccine safety experts (20). Where an ongoing response was required e.g., for large scale vaccine errors, an Incident Management Team was established, which could expand to include other important partners, such as local public health units, quality and safety bodies, and the Commonwealth Vaccine Operations Centre. In addition, specific terms of reference were developed in partnership with the Coroner's Court of Victoria regarding forensic assessment of deaths following COVID-19 vaccines, with creation of pathways to incorporate forensic assessments into SAEFVIC's review of mortality cases.

Internal AESI-specific databases were set up for enhanced monitoring and expanded data collection, designed around clinical workflows. These datasets have formed the basis for additional research examining AESI in the Australian context (21–23), and work is ongoing to integrate them into SAEFVIC's operational database.

## Establishment of specialist immunisation service network

Immunisation clinics in Victoria have traditionally focused on children, however the (initial) adult focus of the COVID-19 program demonstrated the need to expand specialist services for adults. In partnership with the Department of Health, SAEFVIC helped establish an expanded network of Victorian Specialist Immunisation Services (VicSIS), funded by the Victorian Government. These services enable tailored clinical review for individuals experiencing or at risk for AEFI, as well as access to allergy services and vaccination under observation (24, 25). Clinics are staffed by subspecialists, most commonly immunologists and infectious diseases physicians, with dedicated services developed for patients with allergy and immunosuppressed individuals with underlying cancer. SAEFVIC established a central electronic referral hub, to better triage and coordinate appointments, and link with SAEFVIC's surveillance systems (26).

VicSIS clinicians make an important contribution to AEFI reporting, providing detailed case information, follow up of outcomes over time, and advice on subsequent vaccination doses. Regular meetings between VicSIS clinicians, SAEFVIC, and the Department of Health enable case discussion and knowledge sharing and development of clinical guidelines to support immunisation providers across Victoria. Up to 31 July 2022, VicSIS has conducted 12,288 pre-vaccination and post-vaccination consultations (8). Funding constraints and reduced service demand have led to a reduction in VicSIS services beyond mid-2022. However, VicSIS offers a potential framework for all vaccines, to increase access for patients who have experienced AEFI to receive expert advice and care.

## Vaccine safety communication, education, and training

The Melbourne Vaccine Education Centre (MVEC), based within SAEFVIC, was formed in 2014 (27). In the past year, its website was viewed 1.1 million times. It aims to provide up-to-date authoritative immunisation information for health providers and the public. As well as a suite of online information pages, MVEC hosts regular clinical vaccinology updates for providers and continues to develop online training modules addressing aspects of vaccine administration and safety for immunisers. Data regarding trending vaccine safety concerns, obtained through active social media listening machine learning methods, are also now being utilised to inform MVEC's content generation for the public.

In September 2021, SAEFVIC initiated a weekly public-facing vaccine safety report, modelled on the Public Health Agency of Canada's report, where numbers of overall AEFI reports and a detailed breakdown of AESI reports are updated weekly (8, 28). This provides an important interface to communicate vaccine safety data in a visual, easy-to-read way, that can be quickly be updated to include emerging AEFI and link to appropriate advice.

## Collaboration with partners

Since inception, SAEFVIC has maintained close links with local and national government and regulatory bodies, as well as serving as a key member of vaccine-related clinical collaborations such as the Adverse Events Following Immunisation-Clinical Assessment Network and AusVaxSafety. During the COVID-19 pandemic, collaboration with both existing and new partners and stakeholders has become crucial.

For example, the early emergence of the AESI TTS associated with the Vaxzevria vaccine highlighted the critical importance of specialist networks (e.g., the Thrombosis and Haemostasis society of Australia and New Zealand) in being able to improve case detection, rapidly articulate and disseminate clinical guidance, and facilitate early diagnosis and access to treatment (29). Partnering with haematologists, allergist/immunologists, cardiologists, neurologists, forensic pathologists, and other relevant specialist groups has been key in responding to AESI. Clinical specialists have provided input into assessment of individual cases, contributed to clinical guidelines and case definitions (including Brighton Collaboration definitions), and supported further vaccine safety research.

Additionally, given the rare nature of some AESI, sharing data nationally and internationally can help to improve understanding of underlying biological mechanisms and risk factors. The International Network of Special Immunisation Services is an example of a newly formed international collaboration to study AESI, of which SAEFVIC is the lead Australian member (30). The Global Vaccine Data Network's US Centres for Disease Control funded Global COVID-19 Vaccine Safety study combines de-identified data analyses across 18 countries to explore vaccine associations with rare AESI, with SAEFVIC being one of two Australian partners (31).

At a local level, utilising a harmonised database system with Western Australia (Western Australian Vaccine Safety Surveillance) has allowed shared innovations and collaboration. The vaccine surveillance service of Australian Capital Territory is also utilising SAEFVIC's database framework. Other Australian jurisdictions (or sub-jurisdictional health services) have developed their own vaccine surveillance systems, with lessons learned shared through scientific fora such as the National Communicable Diseases and Immunisation Conference.

Finally, in light of the rapid changes in vaccine policy and expansion of SAEFVIC's work over the pandemic, maintaining internal communication and cooperation has also been important. A weekly SAEFVIC “huddle” became a valuable touch point for staff to hear updates, share information and resources, co-ordinate activities, and support colleagues. As for many workplaces, the backdrop of lockdown requirements necessitated the transition to working remotely for many SAEFVIC staff, making clear communication all the more important.

## Conclusion

Post-licensure vaccine safety monitoring, through both spontaneous and active mechanisms, is a critical component of vaccine program implementation. The COVID-19 vaccine program has brought both immense challenges and unique opportunities for improving vaccine safety science and systems. Throughout the COVID-19 vaccine program, SAEFVIC's work has enabled early detection and better understanding of less frequent AEFI, and contributed safety data to support vaccine policy. It has supported vaccine confidence in Victoria, and facilitated support and follow up for individuals who experience AEFI. Victoria's expanded vaccine safety response has been enabled by having a robust vaccine safety system already in place, drawing on considerable vaccine safety experience and established partnerships. Additional resourcing to expand capacity was critical, and this now presents sustainability challenges for many of the innovations described.

Supporting the COVID-19 vaccine program has required rapid adaptation to frequent vaccine policy changes and evolving vaccine safety signals, careful prioritisation, informatics innovation, and enhanced engagement with the public regarding vaccine safety. Moving forward, with support from the Victorian Department of Health, SAEFVIC is commissioning a new eHealth database infrastructure with increased capacity, flexibility, and the ability to interact with complementary datasets. Long-term investment to continue to strengthen vaccine safety systems, and build on lessons learned, will be essential for the ongoing success of COVID-19 and other vaccine programs into the future.

## Data availability statement

The original contributions presented in the study are included in the article/Supplementary material, further inquiries can be directed to the corresponding author.

## Author contributions

NC and JB conceived the article. All authors were involved in drafting, editing, and reviewing the article.

## Conflict of interest

The authors declare that the research was conducted in the absence of any commercial or financial relationships that could be construed as a potential conflict of interest.

## Publisher's note

All claims expressed in this article are solely those of the authors and do not necessarily represent those of their affiliated organizations, or those of the publisher, the editors and the reviewers. Any product that may be evaluated in this article, or claim that may be made by its manufacturer, is not guaranteed or endorsed by the publisher.
