# Supplementary figures and images for: Vaccine safety in Australia during the COVID-19 pandemic: Lessons learned on the frontline
Source: Front Public Health. 2022 Nov 4;10:1053637. doi: 10.3389/fpubh.2022.1053637 (PMC9672672; doi:10.3389/fpubh.2022.1053637)

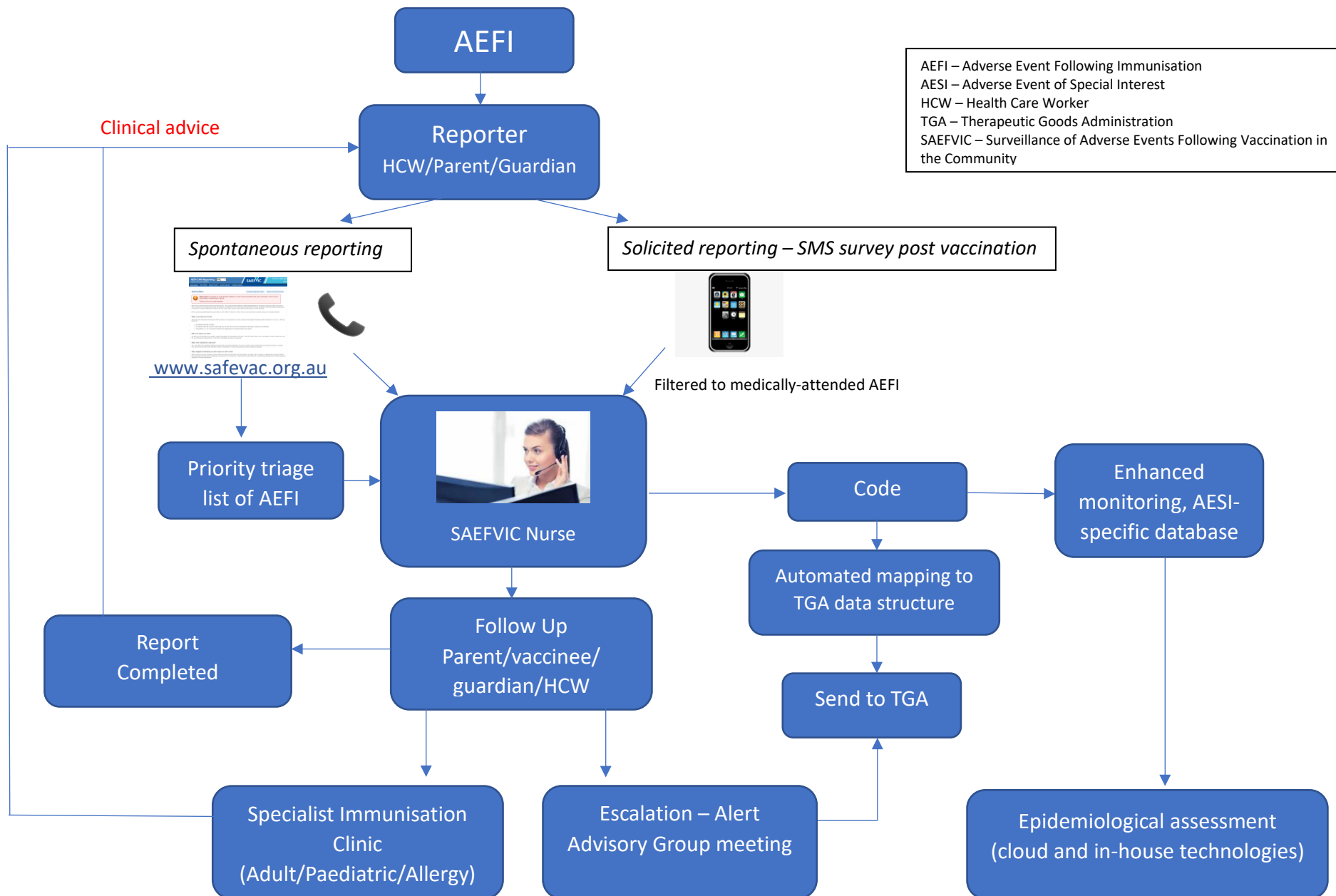

**Figure 3: Summary of SAEFVIC workflow**

Supplement: Supplementary file 1 [file Image_1.pdf]
